# How to Break through the Bottlenecks of in Ovo Vaccination in Poultry Farming

**DOI:** 10.3390/vaccines12010048

**Published:** 2023-12-31

**Authors:** Xuefeng Li, Xiaoxiao Liu, Lu Cui, Zheyi Liu, Yu Zhang, Hai Li

**Affiliations:** 1 Department of Pathogenic Microbiology and Immunology, School of Basic Medical Sciences, Xi’an Jiaotong University, Xi’an 710061, China; lixuefeng@xjtu.edu.cn (X.L.); liuxiaoxiao12@stu.xjtu.edu.cn (X.L.); 2 Key Laboratory of Environment and Genes Related to Diseases, Ministry of Education, Xi’an 710061, China; 3 Division of Avian Infectious Diseases, State Key Laboratory of Veterinary Biotechnology, National Poultry Laboratory Animal Resource Center, Harbin Veterinary Research Institute, the Chinese Academy of Agricultural Sciences, Harbin 150069, China; cuilu099@163.com (L.C.); liuzy0701@126.com (Z.L.)

**Keywords:** in ovo vaccination, vaccines, poultry disease, adaptive immunity

## Abstract

Poultry farming is one of the pillar industries of global animal husbandry. In order to guarantee production, poultry are frequently vaccinated from the moment they are hatched. Even so, the initial immunity of chicks is still very poor as they are in the “window period” of immune protection. In ovo vaccination pushes the initial immunization time forward to the incubation period, thereby providing earlier immune protection for chicks. In ovo vaccination is currently a research hotspot of poultry disease prevention and control, which is in line with the intensification of poultry production. However, the vaccines currently available for in ovo vaccination are limited and cannot meet the needs of industrial development, so how to efficiently activate the adaptive immune response of chicken embryos becomes the key to restrict product development and technological progress of in ovo vaccination. Its breakthrough, to a large extent, depends on systematic illustration of the mechanism underlying the adaptive immune response post immunization. Clarification of this issue will provide us with theoretical support and potential solutions for the development of novel vaccines for in ovo vaccination, the augmentation of efficacy of current vaccines and the optimization of immune programs.

## 1. Introduction

As a major poultry production country, China has a long history of poultry farming, with the second-ranking position in global poultry meat production and first-ranking position in global egg production [1,2,3]. During the breeding process, poultry are susceptible to various factors such as the rearing environment, operational techniques, nutritional conditions and pathogenic microorganisms, thereby reducing production traits and causing economic losses. In order to guarantee production, poultry are frequently subjected to vaccination from the moment they are hatched. Nevertheless, the immune defense ability of chicks within the first two weeks of hatching is still poor and they are highly susceptible to various pathogens. This is because the newly hatched chicks usually have not been exposed to external pathogens before hatching, and even if vaccinated immediately after hatching, it is still the initial immunization, requiring approximately two weeks for body to establish an adaptive immune protection. During this period, young chicks only rely on limited maternal antibodies to resist external pathogens; thus, they are reckoned in a “window period” of immune protection.

In order to address the above issue, researchers have endeavored to move the initial vaccination time in poultry forward to the incubation period by carrying out chicken embryo immunization, also known as in ovo vaccination. By vaccinating chicken embryos at the later stage of hatching, this method enables chicks to gain a certain level of immunity before or shortly after hatching, thereby reducing or blocking the invasion of specific pathogens. The advantages of in ovo vaccination are reflected in the following: (1) in ovo vaccination is able to induce the body’s adaptive immune response earlier, thus shortening the “window period” of immune protection in chicks; (2) in ovo vaccination reduces the frequency of chicken immunization; (3) by adopting an automated platform, in ovo vaccination enhances the precision and uniformity of immunization, improving the efficacy of vaccine administration; and (4) in ovo vaccination effectively avoids the stress reaction and death of chicks during vaccination, thereby improving the survival of chicks [4].

After more than three decades of development, in ovo vaccination has emerged as the preferred vaccination method in several leading poultry-producing nations, including the United States, Brazil, nations in the European Union, Japan, and Argentina. This technology has also been embraced on a smaller scale by numerous other countries, such as Turkey, nations in the Middle East, and other Latin American countries. Presently, the adoption rate of in ovo vaccination in the global poultry farming industry has reached 32%, and in the United States, more than 90% of broiler chickens receive their initial immunization through in ovo vaccination [5]. With the significant increase in the intensification level of poultry farming in developing countries in recent years, in ovo vaccination technology will enter a period of rapid development in the coming years. Notwithstanding that in ovo immunization is a future direction of disease prevention and control in the poultry industry, only a limited number of in ovo immunization vaccines have been approved for production and widely used, which, to a large extent, restricts the promotion of in ovo vaccination technology [6].

In this review, we summarized the research progress and current applications of in ovo vaccination technology and pointed out the bottlenecks restricting the development and technological advancement of in ovo vaccination products. The application of the big-data-driven research paradigm to study the in ovo immunization mechanism will bring us new opportunities to break through the bottlenecks, providing potential solutions for the development of new vaccines, augmentation of efficacy of current vaccines, and optimization of immune programs.

## 2. Principles and Methods of in Ovo Vaccination

### 2.1. Physiological Basis for in Ovo Vaccination

As a classical visualization model of in vitro development, chicken embryos have been widely used in basic scientific research, such as developmental biology, embryology and immunology. The immune system is a complex regulatory system composed of a series of tissues, organs, cells and even cellular factors, which undergoes continuous development and gradually forms throughout the embryonic and fetal development process. Among the poultry immune system, the thymus is the earliest formed lymphoid organ. Previous research shows that the fully matured structure of the chicken embryo thymus forms on the 18th day of incubation, while the result from lymphocyte function testing suggests that the lymphocytes within the thymus exhibit an anti-host graft response as early as two weeks from chicken embryo hatching. This finding indicates that the thymus lymphocytes already possess a certain level of immune activity at this time [7]. In the meanwhile, B lymphocytes, which are responsible for mediating the body’s humoral immunity, mature in the bursa of Fabricius of chicken embryos [8]. Thus, it can be seen that the adaptive immune system of chicks is almost matured before they are hatched from the shell. During this period, in ovo vaccination will be carried out to activate the lymphocytes in chicken embryos, thereby facilitating them to establish the corresponding immune protections as early as possible [9].

### 2.2. Principles and Methods of in Ovo Vaccination

**Figure 1:**
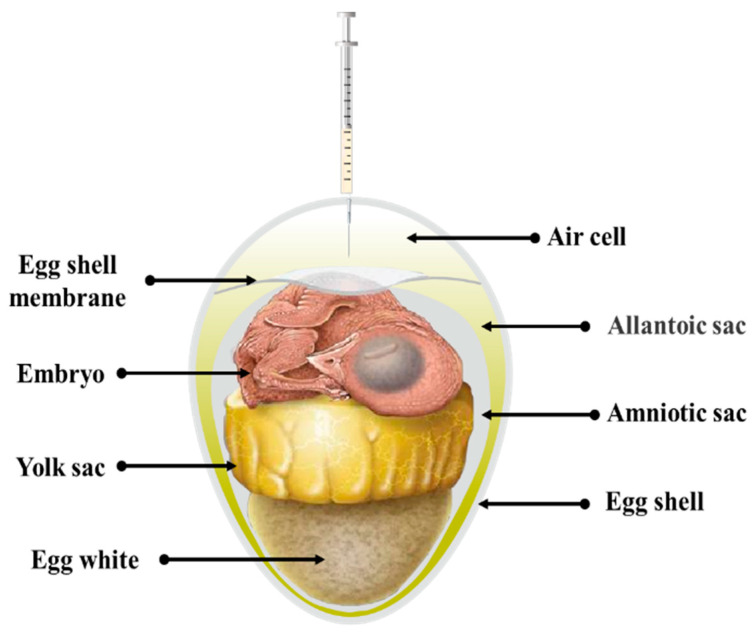
Components of breeding egg after 18 days of hatching.

In practical production, in ovo vaccination is performed during the transfer of breeding eggs from the incubator to the hatching chamber. Although the chicks have almost formed at this time, we also need to pay attention to the operation, avoiding embryo damage and pathogenic infection. Chicken embryos aged 18–19 days are usually used for in ovo vaccination, when the internal space of eggs is nearing capacity and the majority of the yolk sac has been assimilated into the abdominal cavity (Figure 1). To ensure sterile conditions for vaccination, the eggs are carefully wiped with iodine and alcohol, and then a hole is drilled in the eggshell above the air cell. In ovo vaccination is carried out using 18 G or 22 G needles, with a needle length of approximately 25.4 mm. The needle is vertically inserted through the allantoic sac into the amniotic cavity, and the vaccine is injected into the neck or the shoulder back of chicken embryos, with a maximum dose of 0.05 milliliters. After vaccination, the perforations are sealed with wax oil or iodine-sterilized tape and continue to hatch until the chicks reach 21 days old.

To conduct in ovo vaccination, accurate controls should be exercised over the embryonic development stage, vaccine preparation and delivery conditions as well as the biosafety level of the incubation plant, because the satisfactory immunization outcome necessitates rigorous quality control measures. In addition, the needles used for in ovo immunization must be strictly disinfected to avoid contaminating the chicken embryos during vaccination, therefore affecting their normal development.

## 3. Development and Applications of in Ovo Vaccination Technology

### 3.1. Applications of in Ovo Vaccination Technology

In 1982, Sharma and Burmester discovered that inoculation of chicken embryos with turkey herpesvirus on the 18th day of hatching could effectively protect the hatched chicks from Marek’s disease virus infection [10]. This study, for the first time, showed that chicken embryos are able to establish an adaptive immune response when stimulated by an external antigen, thereby shortening the “window period” of immune protection upon similar antigen exposure after hatching. In the 1990s, in ovo vaccination technology gradually became commercialized and quickly gained widespread application in more than 30 countries including the United States. This led to a gradual shift in chicken breeders from the traditional immunization of one-day-old chicks to the more advanced and safer in ovo vaccination [11]. In order to meet the needs of industrial development of in ovo vaccination, in 1992, Embrex Inc. (belonging to Zoetis Animal Health Inc., NJ, USA) in the United States developed the first commercial injection device for in ovo vaccination, namely Inovoject, which enabled the immunization of 30,000 chicken embryos with the assistance of two workers per hour [12]. Since then, Zoetis has consistently held its position as a leading provider of in ovo vaccination technology. At present, the company offers two primary products: the fully automated Inovoject, also known as the Inovoject NXT system, and the semi-automated Inovoject model M [13]. The Inovoject NXT can process up to 70,000 eggs per hour and features dual-needle technology for precise injection. The Inovoject model M, designed for smaller hatcheries, handles between 12,000 and 20,000 eggs per hour, which allows smaller hatcheries to reap the benefits of in ovo vaccination in a more compact form. In addition to Zoetis, other companies like Innovatec and Ceva also offer mechanized systems for in ovo vaccination. Innovatec’s Ultimate In-Ovo system optimizes biosecurity and productivity with automatic needle cleaning and pre-vaccination egg removal [14]. Ceva’s Egginject^®^ system, adaptable to all incubation trays, adjusts the injection depth for each embryo and can process up to 60,000 eggs per hour [15]. These mechanized systems enhance the accuracy, reliability and efficiency of in ovo vaccination.

The United States, Brazil, China, and the European Union, as the top four poultry-producing regions, contribute to approximately 60% of the total global chicken meat production in 2022 and 2023 [16]. Apart from China, these countries also represent the three largest in ovo areas in the world [17]. Presently, in ovo vaccination is employed in over 90% of broiler chickens in the United States, and the proportion of in ovo immunization in European broiler chickens has also reached over 40% [5]. In Brazil, the application of in ovo vaccination began in 1999, and by 2014, an annual count of around 4 billion eggs were being vaccinated [18]. Compared with the United States, Brazil, and European countries, the overall development of China’s poultry industry is relatively backward. Until 2017, in the United States, more than 60 laying chicken companies with a stock of over 1 million produced 87% of eggs, including 17 companies with a stock of over 5 million, while in China, 68% of eggs came from retail farmers who raised less than 5000 chickens [6]. The situation of broiler farming was similar. Thus, the professional level of chicken practitioners in China was uneven, and it was once difficult to carry out in ovo immunization. In recent years, with continuous improvement of the intensive production level of China’s poultry industry, some large leading enterprises have gradually carried out in ovo vaccination for disease prevention and control.

### 3.2. Research Progress of in Ovo Vaccination Technology

At present, Marek’s disease (MD) and infectious bursal disease (IBD) vaccines are the most widely used in ovo vaccines in the market. In 2006, Boehringer Ingelheim introduced Vaxxitek^®^HVT+IBD, the first commercially available bivalent recombinant vaccine designed for in ovo vaccination. This innovative vaccine simultaneously safeguards chicks against both MD and various pathotypes of the IBD virus by expressing the VP2 protein of IBD virus using turkey herpes virus (HVT) as the vector [19]. The vaccine proved to be a tremendous success, with sales exceeding 130 billion doses. Based on this, researchers have developed a range of bivalent recombinant vaccines for in ovo vaccination, such as chimeric recombinant Newcastle disease (ND) vaccine. Administration of this vaccine stimulates the body to produce antibodies against both Newcastle disease virus (NDV) and infection bursal disease virus (IBDV), thereby conferring immunoprotection against these viruses simultaneously [20]. Recombinant vaccines that utilize other viral vectors also exist. For instance, Ceva offers Vectormune^®^FP poultry vaccines, which employ the fowl poxvirus (FP) as a vector [19]. In 2019, Kou et al. conducted a study employing HVT-IBDV live vector vaccine to immunize white-feathered broiler chickens. Their findings showed that after 28 days of vaccination, the immunoprotection rates of an in ovo immunization group and one-day old immunization group were both 100%, but compared with traditional subcutaneous injection, in ovo immunization exhibited superior performances in terms of epidemic prevention cost and immunization speed [21]. The first trivalent in ovo vaccine, the Vaxxitek^®^HVT+IBD+ND vaccine, was also provided by Boehringer Ingelheim in 2019. This vaccine offers protection against MD, IBD, and ND simultaneously. In 2020, their second trivalent vaccine, Vaxxitek^®^HVT+IBD+ILT, received marketing authorization in the United States, providing protection against three diseases with just one dose. Currently, several companies offer commercial trivalent in ovo vaccines, including INNOVAX-ND-IBD and INNOVAX-ND-ILT from Merck and HVT-IBDV-LT from FARVET. These bivalent and trivalent in ovo vaccines offer a convenient and cost-effective solution for preventing highly contagious and commercially destructive diseases that impact the global poultry industry. The effectiveness of these vaccines in safeguarding against infectious poultry diseases and enhancing flock performance and productivity has been extensively documented in prior research [20,22].

In addition, researchers also commit to optimizing compositions of in ovo vaccination vaccines and vaccination methods. Lillehoj et al. mixed recombinant Eimeria profilin with Clostridium Perfringens NetB protein, and the mixtures fortified with or without Montanide^TM^ vaccine adjuvant were used to inoculate 18-day old chicken embryos to compare their immunoprotection effects against necrotizing enterocolitis [23]. The results showed that the vaccine adjuvant-added group was better than the non-adjuvanted group in terms of average body weight gain, reduced intestinal damage and increased antibody level, indicating that the addition of appropriate adjuvants can also enhance the immunoprotection effect of in ovo vaccination [23]. Ju et al. investigated the efficacy of in ovo injection of Newcastle disease vaccine adjuvanted with lactic acid bacteria in specific pathogen-free eggs on day 18.5 of incubation, and the result showed that this treatment has a positive effect on the growth performance, immune function and microbiome of growing chicks [24]. Gaghan et al. performed in ovo adjuvantation of recombinant herpesvirus of turkey-Laryngotracheitis (rHVT-LT) vaccine with CpG-oligonucleotides (ODN), an agonist of avian toll-like receptors 21, and they found that CpG-ODN could be used as an effective adjuvant for rHVT-LT in ovo vaccination to induce immunity against infectious laryngotracheitis in broiler chickens [25]. In addition, Rauw et al. used recombinant turkey herpesvirus expressing NDV-F gene for in ovo immunization of laying hens, and on the first day of hatching, the chicks were revaccinated with chitosan-adjuvanted ND vaccine. This novel inoculation method induced an elevated level of immune response and significant reduction in virus shedding [26].

From the above studies, we can see that constructing polyvalent recombinant vaccines for in ovo immunization and optimizing vaccine composition and vaccination method will not only improve the in ovo vaccination efficiency but will also minimize the stress-induced damage resulting from repeated vaccination in chickens.

## 4. Bottlenecks in the Development of in Ovo Vaccination

Although in ovo immunization is a hot topic and future research direction of disease prevention and control in poultry industry, only a limited number of in ovo immunization vaccines have been approved for production and widely used currently, including the ones against MD, IBD, ND, FP, and avian coccidiosis (AC) [5]. There are two primary factors contributing to this situation. Firstly, although the currently approved live in ovo vaccines are able to promptly activate adaptive immune response in chicken embryos and effectively shorten the “window period” of immune protection, a part of live virus vaccines, which are typically administered to newly hatched chicks, may impede the development of chicken embryos [27,28]. For instance, the Newcastle disease virus Bl strain [27] and clone-30 [28] are lethal to chicken embryos. Even the former approved in ovo vaccine against AC has been identified to negatively impact the development of chicks in long-term studies [29,30]. Moreover, live vaccine strains can survive and replicate for a long time in the vaccinated individuals, which raises concern regarding their potential biosafety risks, thereby limiting the approval and application of such vaccines. Secondly, inactivated vaccines and genetically engineered vaccines, including recombinant vector, DNA and protein subunit vaccines, have low biosafety risks and rarely affect the development of vaccinated chicken embryos, making them the main research direction for future of in ovo vaccines in poultry industry. However, the immune effects of the above-mentioned vaccines are not satisfactory. For instance, when employing inactivated avian influenza and Newcastle disease vaccines for in ovo vaccination, hemagglutination inhibitory antibodies were generated nearly two weeks after the chick hatching, failing to display the advantage of significantly shortening the immune protection “window period” [31]. Therefore, how to effectively initiate the adaptive immune response in chicken embryos has become the bottleneck restricting the development of in ovo vaccination products and the technological advancement of in ovo immunization.

## 5. How to Break through the Bottlenecks of in Ovo Vaccination

### 5.1. Understanding the Initiation Mechanism of Chicken Embryo Adaptive Immune Response

To regulate and control the adaptive immune response in chicken embryos, we should understand the core scientific issues behind it, namely the mechanisms underlying the initiation of adaptive immune response in chicken embryos.

**Figure 2:**
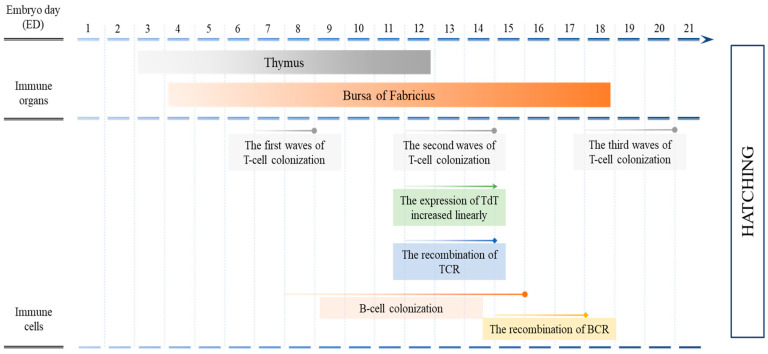
Adaptive immune system development in chicken embryos.

It has been fully proven that mature T lymphocytes and B lymphocytes are the primary effector cells of adaptive immune response in chicken. As shown in Figure 2, the thymus gland, the initially formed immune organ in chicken embryos, commences on the 3rd day of incubation and becomes fully mature by the 12th day [8,32,33]. In contrast, the developmental period of bursa of Fabricius, the avian specific immune organ, is much longer, characterized by the gradual establishment of the lymphoid tissue framework, reticulum, from day 4 to day 18 of incubation [32,34]. Along with the maturation of lymphoid organs, the differentiation of lymphocytes takes place. The presence of T and B lymphocytes can be detected for the first time on approximately the 10th day of incubation [33,35,36]. By day 14 of incubation, both the thymus and the bursa of Fabricius, within which T lymphocytes and B lymphocytes develop and mature, respectively, have formed [37]. There are three developmental peaks of T lymphocytes in chicken embryos, namely day 7, day 12 and day 18 of incubation, each lasting 1–3 days [38,39,40]. The enzyme responsible for the reconstitution of T cell antigen receptors (TCRs), known as terminal deoxyribonucleotidyl transferase (TdT), initiates its expression in the thymus on the 12th day of chicken embryo development and exhibits a linear increase during the second developmental peak of T lymphocytes [41,42]. Correspondingly, the recombination of thymic T cell antigen receptor TCRβ occurs at this stage, suggesting the maturation of T lymphocyte immune function [43,44]. The negative selection of autoreactive thymocytes is achieved before day 14 of incubation, and thereafter, the efficacy of in ovo vaccination is not compromised by immunological tolerance [45,46]. From day 8 to day 15 of chicken embryo hatching, B lymphocytes also colonize in the bursa of Fabricius [33,36,47,48,49]. But later than T lymphocytes, the recombination of B cell antigen receptors (BCRs), based on corresponding pairs of functional variable region heavy and light chain genes, occurs from day 15 to day 17 of chicken embryo hatching [48,50]. Therefore, when immunization is carried out on the 14th day of hatching, it is theoretically possible for T lymphocytes to respond to the stimulations of antigen-presenting cells such as dendritic cells and monocytes, so that the activated T lymphocytes further activate B lymphocytes and cytotoxic T lymphocytes to complete the initiation of adaptive immune response in the chicken embryos before hatching out from the shells. However, several former studies have shown that it is until approximately day 18 of hatching that in ovo vaccination is able to effectively initiate the adaptive immune responses in chicken embryos [51,52]. So, what are the differences in the immune cell composition and their immune response processes between the 14-day-old chicken embryos and 18-day-old ones? This becomes the key point to understand the initiation mechanism of adaptive immune response in chicken embryos.

In order to investigate the variations in cellular composition and immune response process during this interval, researchers have conducted some preliminary explorations on the minute structures of thymus and bursa of Fabricius, lymphocyte composition and molecular expression profiles, etc., via histology, embryology, molecular cell biology and multi-omics [53,54,55,56,57,58]. In 2015, Gimeno et al. found that in ovo immunization with MD live vaccine not only triggered T cell activation but also promoted the maturation of immune system in chicken embryos; however, due to the limitations of the research methods and analytical tools of the day, their study lacked further investigation of the patterns of immune cell change following immune system activation [59]. In addition, there are significant differences in the temporal dynamics of adaptive immune responses induced by different types of in ovo vaccines. For instance, effective immune protection can be triggered 6 days post vaccination with live vaccines on the 18th day of chicken embryo hatching, whereas the activation of adaptive immune response by inactivated vaccines requires more than 10 days [31,52]. Identifying the factors contributing to the discrepancy of immune response will also contribute to the comprehension of initiation mechanism of adaptive immune response in chicken embryos.

### 5.2. Utilizing New Detection Technologies and Developing Suitable Analysis Methods

The vertebrate immune system is complex, and their adaptive immune activation is the interaction result of a large number of innate and adaptive immune cells. In recent years, the big-data-driven research paradigm based on single-cell omics has been widely used in immunological studies pertaining to vaccination, which holds promising implications for the future rational design of vaccines. For instance, Zhao et al. conducted single-cell RNA sequencing on peripheral blood mononuclear cells (PBMCs) from individuals exhibiting high immune response or no immune response to hepatitis B vaccine [60]. Their research elucidates the molecular underpinnings of the non-response to hepatitis B vaccine, which offers potential targets that could be used for enhancing the robustness of immune response to hepatitis B vaccination. This method breaks through the limitations of traditional research methods, enabling the comprehensive exploration of the dynamic alterations in immune cell composition and interaction, thereby expanding our understanding of the intricate nature of immune responses upon vaccination. The application of this novel research paradigm to study in ovo immunization mechanism will bring us new opportunities to break through the bottlenecks restricting the development and technological advancement of in ovo vaccination [61,62].

**Figure 3:**
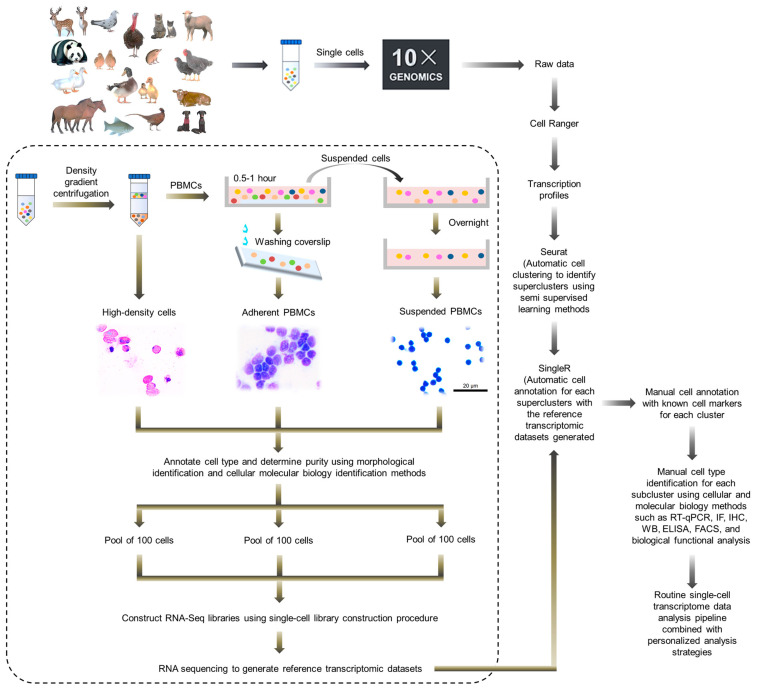
Data processing and analysis flowchart for immune system single-cell transcriptional sequencing of non-model animals.

It should be noted that, among different species, there are differences in the compositions of immune system, the degree of genome annotation and the accumulation of research data regarding immune responses, which brings difficulties for the application of core analysis methods for single-cell omics technology in the non-model animals, such as cell annotation and immune library analysis. For example, in previous studies, we attempted to use homologous genes between avian and human or mice to annotate avian immune cells using existing human and mouse single cell annotation platforms and databases. However, this method can only provide annotations for a portion of avian T lymphocytes with low accuracy. In view of this, in 2021, our laboratory comprehensively applied the physical and morphological characteristics of immune cells, cell molecular markers and semi-supervised learning methods, by means of a hierarchical annotation strategy, and we accurately annotated the main avian immune cells and drew the first single-cell transcriptomic landscape of the non-model animal immune system [63]. The data processing and analysis flowchart is shown in Figure 3, including the following: (1) preliminary separation and recovery of various immune cells are carried out according to their physical characteristics, and identification and purity statistics are conducted using tissue morphology and molecular cell biology methods. By adjusting the cell separation and recovery parameters, ideal purity and integrity are achieved. (2) On the one hand, all single cells sequenced are clustered using semi-supervised learning methods to achieve unbiased annotation of these cells at the first level (supercluster level), and on the other hand, reference transcriptomies for various types of cells are prepared using a single cell library construction scheme. (3) Then, cells are annotated with SingleR using the reference dataset established according to their transcription profiles combined with currently known cell markers to conduct second-level cell annotation (cluster level). (4) Finally, individual subpopulations that cannot be automatically annotated were manually identified and annotated using cell function analysis and comprehensive cellular and molecular biology methods such as reverse transcription quantitative PCR (RT-qPCR), immunofluorescence (IF), immunohistochemistry (IHC), Western blotting (WB), enzyme-linked immunosorbent assay (ELISA), and fluorescence-activated cell sorting (FACS) (subcluster level). After that, the routine single-cell transcriptome data analysis pipeline can be performed in combination with personalized analysis strategies. The analysis results show that this hierarchical annotation strategy can efficiently and accurately annotate avian immune cells, laying the foundation for further single-cell omics detection and data analysis of avian immune responses, such as dynamic changes in immune cell composition, factor secretion, etc., after vaccination or pathogen infection.

Based on this, we have completed the efficient and accurate annotation of duck immune cells after infection with Tembusu virus (TMUV), a mosquito-borne flavivirus newly emerged from ducks with a broad host range from mosquitos to mammals and causing massive economic losses in the Chinese poultry industry. Based on the strategy described in Figure 3, we constructed the transcriptomic landscape of duck immune cells during TMUV infection at single-cell resolution, which is the first single-cell dynamic transcriptional atlas of a non-model animal. By analyzing the process, we unveiled the characteristics and mechanism by which TMUV escapes from the first line of the host antiviral defense system in its arthropod-borne transmission route and revealed the important role of myeloid cells in the early immune response against the virus [63]. In addition to birds, this strategy theoretically can be applied to other non-model animals, such as pets and wildlife. Its universal applicability in practical applications needs to be tested and refined in more single-cell transcriptomic studies on the immune systems of non-model animals.

Since then, there have been increasing publications on the immune system single-cell omics of non-model animals based on similar analysis strategies. From 2022 to 2023, using single-cell RNA-sequencing technology, Dai et al. characterized the T-cell-mediated immune responses of mallard duck upon infection with H5N1-avian influenza virus (H5N1-AIV) and identified the key host factors causing inflammatory lung injury in chickens infected with H5N1-AIV [64,65]. In 2022, the same technology was also used for investigating the transcriptomic landscape of primarily isolated porcine alveolar macrophages upon African swine fever virus (ASFV) infection, which extensively elucidated the pathogenesis of ASFV infection of macrophages in the lung [66], followed by the recent study investigating the responses of different types of cells in the jejunum of piglets after porcine epidemic diarrhea virus (PEDV) infection [67].

As a newly emerging technology, although the findings of current single-cell omics research on immune system and antiviral immune responses of non-model animals are not yet so rich, and since even the related research strategies and analysis methods are still in the exploratory stage, it is not difficult to see the unique advantages of single-cell omics technology in exploring the mechanisms of the complex interactions between hosts and pathogens, especially immunointeraction. Thus, the application of the novel research paradigm to study the in ovo immunization mechanism will bring us new opportunities to break through the bottlenecks restricting the development and technological advancement of in ovo vaccination products.

## 6. Conclusions

As an emerging and efficient vaccination method, in ovo vaccination has received increasing attention in the field of poultry disease prevention and control in recent years. This review provides comprehensive descriptions of the technical principles, methods and strategies, research history and current applications of in ovo vaccination in poultry farming. It also points out that the efficient initiation of adaptive immune response in chicken embryos post immunization is the key bottleneck that currently restricts new product development and technological progress of in ovo vaccination. Researchers should focus on the core scientific issues to systematically investigate the underlying mechanisms regulating the initiation of adaptive immune response of chicken post immunization and to explore potential intervention strategies, so as to provide potential solutions for the development of new vaccines for in ovo vaccination, augmentation of the efficacy of current available vaccines, and optimization of vaccination programs.

## Data Availability

No new data were created or analyzed in this study. Data sharing is not applicable to this article.
